# Cefazolin-induced seizures in a haemodialysis patient with elevated serum and cerebrospinal fluid concentrations: a case report

**DOI:** 10.1093/jac/dkaf373

**Published:** 2025-10-01

**Authors:** Kohei Hasegawa, Kohei Yamamoto, Yoshiki Yamamoto, Itsuki Okamoto, Mitsuyo Nakano, Yoshihiko Ogawa

**Affiliations:** Department of Infectious Diseases, Sakai City Medical Center, Ebaraji 1-1-1, Sakai, Osaka, Japan; Department of Pharmacology, Sakai City Medical Center, Ebaraji 1-1-1, Sakai, Osaka, Japan; Department of Pharmacology, Sakai City Medical Center, Ebaraji 1-1-1, Sakai, Osaka, Japan; Department of Infectious Diseases, Sakai City Medical Center, Ebaraji 1-1-1, Sakai, Osaka, Japan; Department of Infectious Diseases, Sakai City Medical Center, Ebaraji 1-1-1, Sakai, Osaka, Japan; Department of Infectious Diseases, Sakai City Medical Center, Ebaraji 1-1-1, Sakai, Osaka, Japan

Cefazolin, a first-generation cephalosporin, has traditionally been considered to have limited permeability into the CNS.^1^ However, therapeutic efficacy against CNS infections has been reported.^1,2^ In addition, neurotoxic effects such as seizures have been documented, particularly in patients with renal impairment.^3,4^

To the best of our knowledge, no previous reports have documented CSF concentrations in patients who develop neurotoxicity during cefazolin therapy. Herein, we describe a rare case of cefazolin-induced status epilepticus in a patient with renal dysfunction, in whom both serum and CSF drug concentrations were successfully measured. Written informed consent was obtained from the patient for publication of this case report and the accompanying image in an open-access online publication.

A 72-year-old man with a history of end-stage kidney disease on haemodialysis for 4 years, right cerebellar haemorrhage, and diabetes treated with an insulin analogue was admitted for a foot ulcer. On hospital day 4, cefazolin (2 g/d) was administered for concomitant ulcer infection and continued thereafter.

The patient developed impaired consciousness on day 10 of hospitalization. His level of consciousness was E4V4M6 on the Glasgow Coma Scale. His vitals were blood pressure, 148/70 mm Hg; heart rate, 83 bpm; respiratory rate, 16 breaths/min; and body temperature, 36.5℃. No evidence of hypoglycaemia, electrolyte abnormalities, or other potential causes of impaired consciousness was observed. Laboratory findings revealed an albumin level of 2.9 g/dL, a creatinine level of 7.59 mg/dL and an estimated glomerular filtration rate of 6.19 mL/min/1.73 m^2^. On hospital day 13, generalized seizure-like activity was observed. The patient had no history of seizures. The blood glucose level was 62 mg/dL, but the seizures persisted despite correction of blood glucose. CSF examination revealed a white blood cell count of 12/μL, a protein level of 73.4 mg/dL, and a glucose level of 36 mg/dL. Brain magnetic resonance imaging revealed an old cerebral haemorrhage in the right hemisphere, and intermittent electroencephalography (EEG) revealed high-amplitude rhythmic fast activity involving extensive regions of the right hemisphere. He was diagnosed with symptomatic epilepsy and antiepileptic therapy was initiated. Considering the potential effects of the drug, the administration of cefazolin scheduled for hospital day 13 was withheld, and vancomycin was initiated for the foot ulcer infection.

Subsequently, serum and CSF concentrations of cefazolin were measured. Trough serum levels obtained prior to dialysis were 192.6 and 217.0 µg/mL on hospital days 10 and 13, respectively. The CSF concentration was 18 µg/mL on hospital day 13. Haemodialysis was performed three times per week. Serum cefazolin levels before dialysis were reduced to 46.0 and 36.6 µg/mL on hospital days 15 and 17, respectively. The CSF concentration also declined to 1.6 µg/mL on hospital day 20 (Figure 1). A Naranjo adverse drug reaction probability score of +7 indicated a probable association between the patient’s symptoms and cefazolin-induced neurotoxicity.^5^

**Figure 1.**
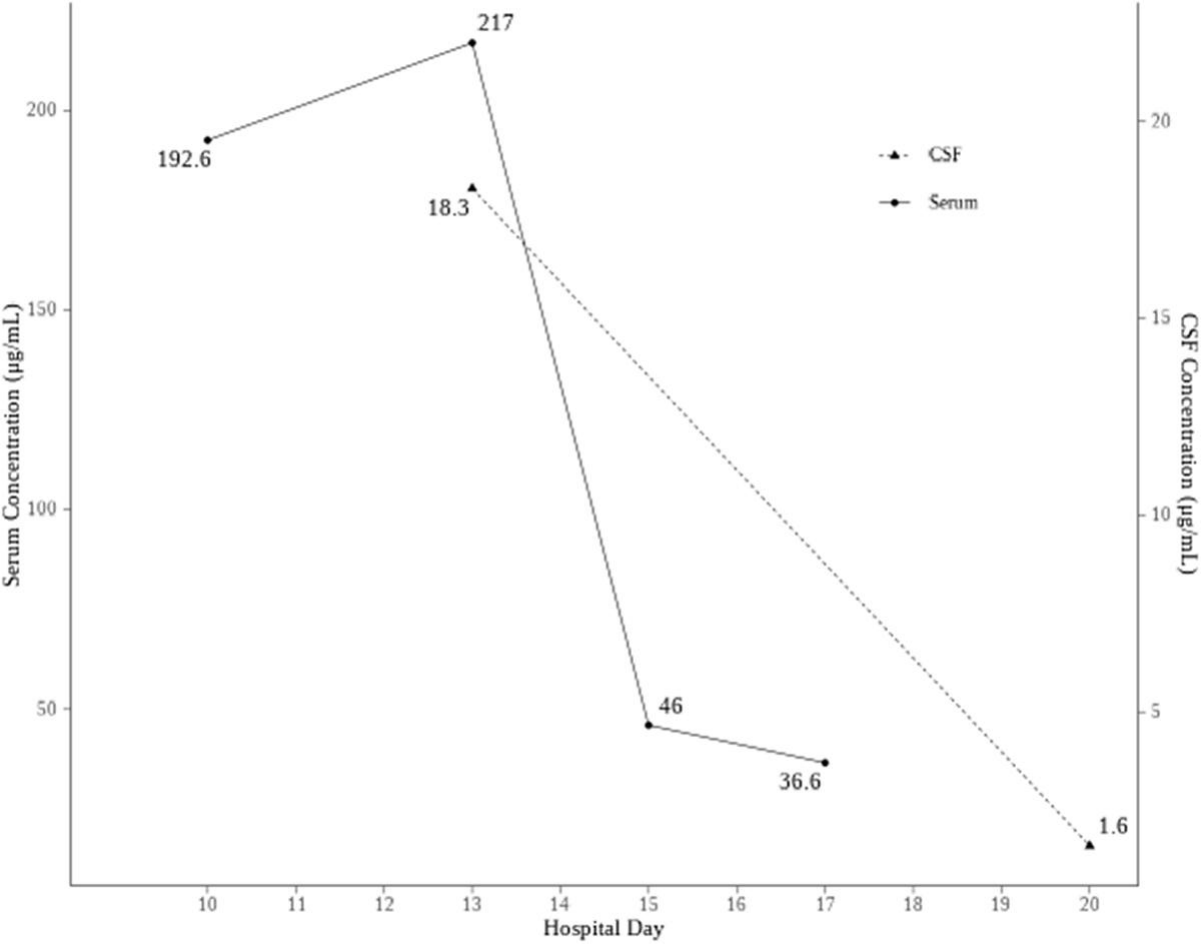
Measured serum and CSF cefazolin concentration. Impaired consciousness started on day 10. The last cefazolin administered on day 12. Dialysis was performed on days 10, 13, 15, 17, and 19. CSF, cerebrospinal fluid.

After cessation of cefazolin and initiation of antiepileptic therapy, the patient’s consciousness gradually recovered. On hospital day 21, antiepileptic therapy was discontinued as the follow-up EEG demonstrated no abnormal findings. No seizure recurrence was observed during follow-up period.

In this case, the cefazolin dose administered was approximately twice the appropriate dose based on the patient’s renal function.^6^ The serum trough concentrations at the onset of impaired consciousness and at the time of seizure were ∼200 µg/mL, exceeding the previously reported level of 149.5 µg/mL in a peritoneal dialysis patient who also developed seizures in the setting of renal impairment.^3^ Recent studies have shown that cefazolin penetrates the CSF more effectively than was previously recognized.^1^ Of particular note is the CSF concentration of 18 µg/mL observed in this case which markedly exceeded median values reported in neurocritical care patients (2.97 µg/mL)^2^ and even in postoperative meningitis patients receiving high-dose therapy of 10 g/d (11.9 µg/mL),^1^ despite the absence of meningitis. To the best of our knowledge, no previous study has measured CSF concentrations in patients who developed neurotoxicity during cefazolin therapy, making this case valuable for understanding the underlying pathophysiology.

Renal impairment was the primary factor contributing to markedly elevated cefazolin concentrations. Since cefazolin is predominantly excreted unchanged via the kidneys, renal dysfunction significantly prolongs its half-life, leading to drug accumulation.^7,8^ Notably, the hypoalbuminaemia observed in this case was considered to have played a contributory role. A reduction in protein binding increases the unbound fraction of cefazolin in plasma, thereby facilitating its penetration into the CNS. Second, the half-life of cefazolin in the CSF (6.5 h) is longer than that in the serum, potentially resulting in prolonged neurotoxicity.^9^ The coexistence of these two factors is presumed to have synergistically created a condition conducive to CNS toxicity.^3^

Haemodialysis may be an effective treatment for cefazolin-induced central neurotoxicity, because cefazolin is efficiently cleared by haemodialysis.^2^ Although the initiation of antiepileptic therapy was a potential confounding factor in this case, the patient’s level of consciousness improved following drug removal via haemodialysis, suggesting that the treatment was likely effective.

The incidence of cefazolin-associated neurotoxicity remains unclear. Elevated serum trough and CSF concentrations may have increased this risk, potentially owing to excessive dosing used in this case. Future studies are warranted to determine the serum and CSF thresholds associated with neurotoxicity and to identify the major risk factors.

## References

[1] McCreary EK, Johnson MD, Jones TM et al Antibiotic myths for the infectious diseases clinician. Clin Infect Dis 2023; 77: 1120–5. 10.1093/cid/ciad357 37310038

[2] Novak AR, Krsak M, Kiser TH et al Pharmacokinetic evaluation of cefazolin in the cerebrospinal fluid of critically ill patients. Open Forum Infect Dis 2021; 9: ofab649. 10.1093/ofid/ofab649 35111872 PMC8802796

[3] Chen CC, Lee DJ, Lin SH. Reversible cefazolin-induced status epilepticus in a peritoneal dialysis patient. Toxicol Rep 2022; 9: 1950–2. 10.1016/j.toxrep.2022.10.011 36561953 PMC9764244

[4] Kingra K, Ariano RE, Sharma A. Seizures associated with high-dose cefazolin in a patient with renal dysfunction: a case report. J Pharm Pract 2025: 8971900251326735. 10.1177/08971900251326735 PMC12518881 40073444

[5] Naranjo CA, Busto U, Sellers EM et al A method for estimating the probability of adverse drug reactions. Clin Pharmacol Ther 1981; 30: 239–45. 10.1038/clpt.1981.154 7249508

[6] Duke C, Parker SL, Zam BB et al Population pharmacokinetics of unbound cefazolin in infected hospitalized patients requiring intermittent high-flux haemodialysis: can a three-times-weekly post-dialysis dosing regimen provide optimal treatment? J Antimicrob Chemother 2024; 79: 2980–9. 10.1093/jac/dkae318 39255245 PMC11531813

[7] Haddad N, Carr M, Balian S et al The blood-brain barrier and pharmacokinetic/pharmacodynamic optimization of antibiotics for the treatment of central nervous system infections in adults. Antibiotics (Basel) 2022; 11: 1843. 10.3390/antibiotics11121843 36551500 PMC9774927

[8] Grill MF, Maganti R. Cephalosporin-induced neurotoxicity: clinical manifestations, potential pathogenic mechanisms, and the role of electroencephalographic monitoring. Ann Pharmacother 2008; 42: 1843–50. 10.1345/aph.1L307 19033476

[9] Pitcock C, Burgess DS, Olney KB. Optimizing cefazolin dosing for central nervous system infections: insights from population pharmacokinetics and monte carlo simulations. Antimicrob Agents Chemother 2025; 69: e0185724. 10.1128/aac.01857-24 40391958 PMC12217449

